# Stimulation Induced Electrographic Seizures in Deep Brain Stimulation of the Anterior Nucleus of the Thalamus Do Not Preclude a Subsequent Favorable Treatment Response

**DOI:** 10.3389/fneur.2018.00066

**Published:** 2018-02-19

**Authors:** Tommi Nora, Hanna Heinonen, Mirja Tenhunen, Sirpa Rainesalo, Soila Järvenpää, Kai Lehtimäki, Jukka Peltola

**Affiliations:** ^1^Faculty of Medicine and Life Sciences, University of Tampere, Tampere, Finland; ^2^Department of Clinical Neurophysiology, Medical Imaging Centre and Hospital Pharmacy, Pirkanmaa Hospital District, Tampere, Finland; ^3^Department of Medical Physics, Tampere University Hospital, Medical Imaging Centre, Pirkanmaa Hospital District, Tampere, Finland; ^4^Department of Neurosciences and Rehabilitation, Tampere University Hospital, Tampere, Finland

**Keywords:** deep brain stimulation, anterior nucleus, thalamus, neuromodulation, epilepsy, electroencephalography

## Abstract

Deep brain stimulation (DBS) of the anterior nucleus of the thalamus (ANT) is a method of neuromodulation used for refractory focal epilepsy. We report a patient suffering from drug-resistant epilepsy who developed novel visual symptoms and atypical seizures with the onset of ANT-DBS therapy. Rechallenge under video electroencephalography recording confirmed that lowering the stimulation voltage alleviated these symptoms. Subsequent stimulation with the initial voltage value did not cause the recurrence of either the visual symptoms or the new seizure type, and appeared to alleviate the patient’s seizures in long-term follow-up. We therefore hypothesize that the occurrence of stimulation induced seizures at the onset of DBS therapy should not be considered as a failure in the DBS therapy, and the possibility of a subsequent favorable response to the treatment still exists.

## Background

Epilepsy is a significant neurologic disorder which can greatly affect a patient’s quality of life. Epilepsy has a prevalence of up to 1.0% (1), and approximately one-third of patients with epilepsy do not gain sufficient benefit from treatment with antiepileptic drugs (AEDs) (2). Even though some of these cases can be treated with resective surgery or vagal nerve stimulation (VNS), adequate seizure control is still not achieved in all patients. This group of patients must be considered as possible candidates for deep brain stimulation (DBS) therapy. DBS is a method of neuromodulation with the aim to modulate the activity of epileptic brain networks in a way that suppresses epileptic seizures. Although its exact mechanisms remain to be completely understood, DBS has achieved positive treatment results in both animal and human studies (2–16).

The Stimulation of the Anterior Nucleus of the Thalamus for Epilepsy (SANTE) trial was a multicenter double-blinded randomized controlled trial, in which a group of 110 patients received DBS of the anterior nucleus of the thalamus (ANT) (5). In the blinded phase, patients were divided into control and stimulation groups, and only the stimulation group received DBS. After the blinded phase, both groups received stimulation. The SANTE study group reported of two patients who experienced “acute, transient stimulation-associated seizures.” One of these patients is more widely known as the “outlier patient.” This patient experienced a remarkable amount of atypical seizures due to ANT stimulation and the onset of these seizures was evidently related to the stimulation. This patient was labeled as an outlier and eliminated from the statistical analysis. What makes the outlier patient intriguing is the fact that the same stimulation parameters initially causing these atypical seizures later led to a decrease in the patient’s seizure frequency.

We report a case of a patient resembling the outlier of the SANTE trial. During video electroencephalography (vEEG) recording, our patient responded to ANT-DBS therapy with similar occurrence of a new type of seizure as well as distinct visual symptoms. Equally in the current case, the initial unfavorable response evolved into a beneficial treatment outcome for the patient.

## Case Presentation

We present a case of a 30-year-old (at the time of vEEG-monitoring in 2011) male patient. The patient has given written consent for the publication of this case report. Our patient was diagnosed with temporal lobe epilepsy with brief complex partial seizures [focal unaware, as defined by ILAE 2017 classification of seizure types (17)] at the age of 11, with some seizures propagating to secondarily generalized tonic–clonic seizures [focal to bilateral tonic–clonic, ILAE 2017 (17)]. A specific characteristic for the seizures of this patient was the usual occurrence of tonic–clonic seizures associated with waking up in the morning. He also has a history of several episodes of status epilepticus (SE), leading to hospitalization. Seizures were initially defined to be of temporal origin due to early EEG-findings. However, in the context of comprehensive epilepsy surgery evaluation, conducted in 2005, the etiology of epilepsy was found to be bilateral occipital cortical dysplasia. Consequently, epileptogenic zone was determined to be located in the occipital lobe and temporal lobe was defined as the ictal onset zone. The patient trialed numerous AEDs, singly and in combination, which failed to adequately suppress his seizures. VNS from 2005 to 2010 was similarly unhelpful. Due to the location of the known cortical dysplasia, the patient was deemed not a candidate for resective surgery. These circumstances led to consideration of installing DBS-device for our patient, and in November 2010, DBS electrodes (model 3389, Medtronic, Minneapolis, MN, USA) and an internal pulse generator (Activa PC, Medtronic, Minneapolis, MN, USA) were successfully implanted.

**Figure 1:**
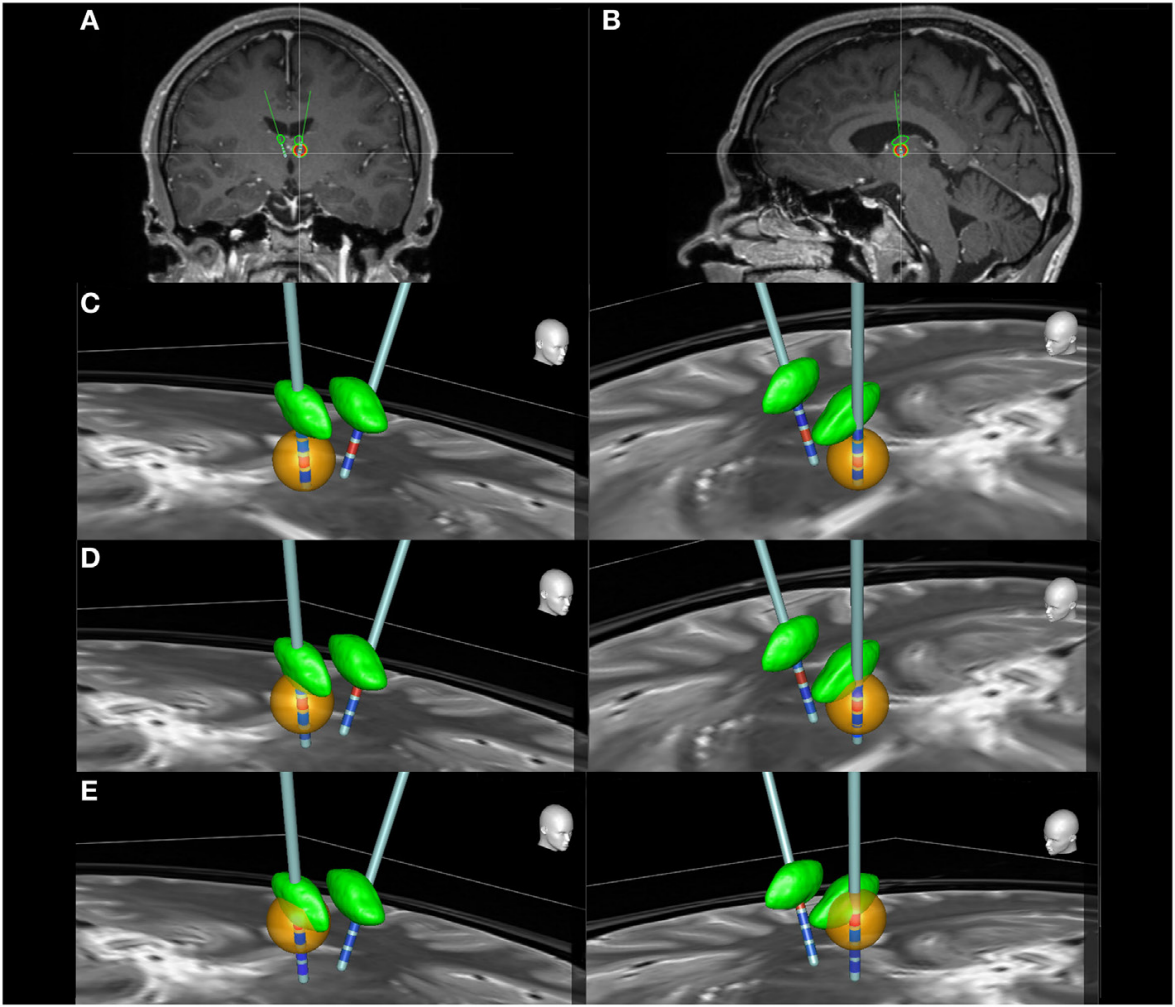
The locations of the deep brain stimulation leads in coronal **(A)** and sagittal **(B)** slices. Panels **(C–E)** illustrate the estimated distribution of the electrical field (orange) generated by different active contacts (red) in both right and left ANT (green). Inactive contacts are represented with blue color. Images were generated using the SureTune software (Medtronic, Minneapolis, MN, USA). From bottom to top, contacts are labeled as 0, 1, 2, and 3 on the left side and as 8, 9, 10, and 11 on the right side. **(C)** Contacts 1 and 9 are active. The electrical field completely misses the ANT. **(D)** Contacts 2 and 10 are active. The electrical field still barely reaches the ANT. **(E)** Contacts 3 and 11 are active. The electrical field extends to the inferior section of ANT. Clinical effect was achieved only with this setting.

The patient’s mean seizure frequency during the five-month baseline period was 12.2 seizures per month, 60 of the 61 seizures being tonic–clonic. Early treatment with ANT-DBS resulted in minor—but temporary—decrease in the seizure frequency, possibly due to microlesion effect described in certain studies (3, 10). Original stimulation parameters immediately after the surgery were set to 5 V amplitude, 90 µs pulse width, 140 Hz frequency, and 1 min ON 5 min OFF cycle. Contacts labeled 2 and 10 were initially active (Figure 1). Voltage was gradually increased to 7 V. As the effect of microlesion began to recede and the patient’s seizures returned to baseline values, active contacts were switched to contacts 1 and 9 (Figure 1). Again, voltage was first set to 5 V and then increased to 7 V. Other parameters were maintained at their original values. Occurrence of a SE episode shortly after this adjustment indicated that these stimulation parameters were not optimal, and the stimulator was shut down temporarily.

**Figure 2:**
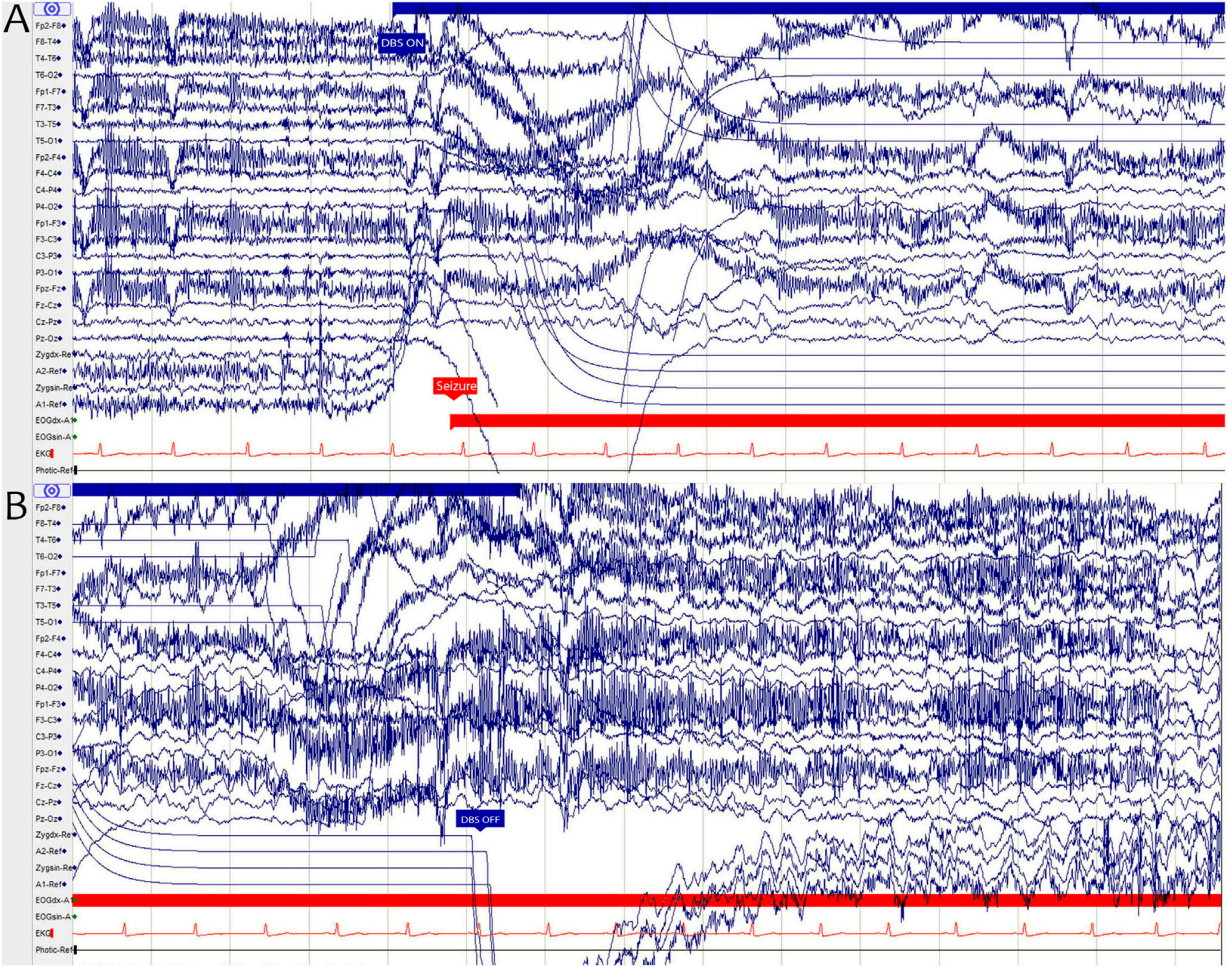
**(A)** The onset of a deep brain stimulation (DBS) ON period (blue arrow) and the onset of an epileptic seizure (red arrow) shown in an EEG graph. **(B)** EEG graph illustrating how the ictal activity continues even after the end of DBS ON period (blue arrow). The sharp “drop” seen in the graph in some channels is an artifact caused by the monopolar cathodal stimulation.

In April 2011, the patient was called in for a 3-day vEEG-monitoring to determine the optimal stimulation parameters in a controlled setting. At the time, his daily medication was set to 1,200 mg of carbamazepine and 30 mg of clobazam. DBS device was switched on during the second day, and stimulation parameters were set to 5 V, 90 µs, 180 Hz, and 1 min ON 5 min OFF—with contacts 3 and 11 being active (Figure 1). Shortly after, at the onset of stimulator ON phase, the patient reported a transient visual symptom consisting of “fogginess” of vision, simultaneously with the onset of ANT stimulation without any abnormalities in the EEG pattern. Later the same day, the onset of stimulator ON phase simultaneously provoked seizure activity clearly seen in the EEG (Figure 2). Clinical manifestations of this seizure differed from the patient’s habitual seizures: The patient was seemingly able to continue his conversation with his roommate, but when the nurse interviewed the patient, he complained that colors seemed different and that the text on a magazine was distorted. After the settling of the seizure, the patient claimed to have no memory of his discussion with the nurse. During the third day, the patient reported several visual symptoms including multiple black to transparent shapes appearing and traveling in his field of vision.

**Figure 3:**
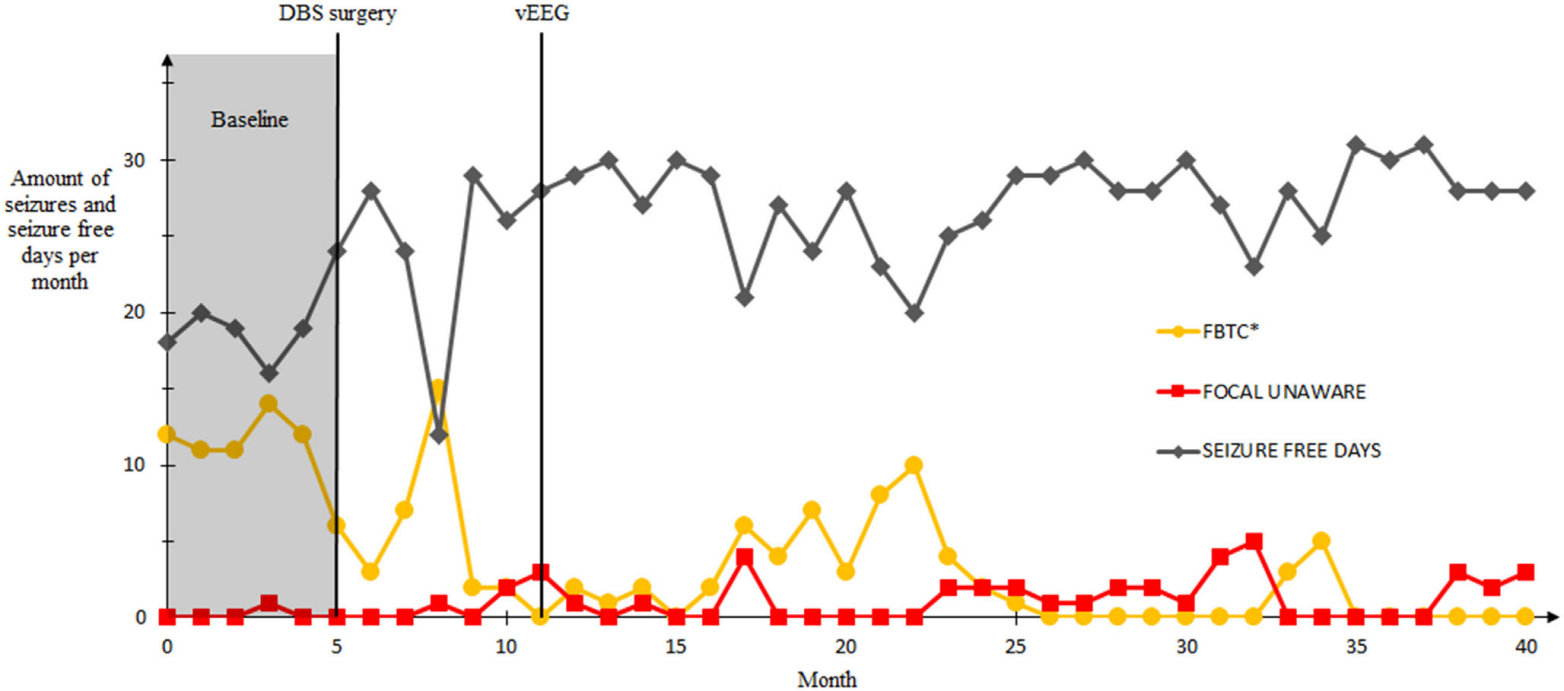
Seizure frequencies and the amount of seizure free days before and after the deep brain stimulation (DBS) surgery in a follow-up of 40 months. First five months represent the baseline seizure frequency (gray area), DBS-surgery was conducted on month 5. The vEEG-recording was conducted on month 11. Seizures that occurred during the video electroencephalography not included. Seizure classification according to ILAE 2017 guidelines. *Focal to bilateral tonic–clonic.

All symptoms vanished as the stimulation voltage was decreased to 3 V during the third day of the recording. The voltage was later that day returned to the value of 5 V and in long-term stimulation was increased up to 7 V, uneventfully. In fact, our patient had long seizure free periods and his overall seizure frequency decreased significantly: During the last 5 months of a 40-month follow-up, our patient had a mean of 1.6 seizures per month. In addition, most of our patient’s seizures during later ANT-DBS therapy were his habitual focal unaware seizures instead of tonic–clonic seizures, which indicates that DBS prevented seizure propagation and reduced seizure severity (Figure 3).

## Discussion

Atypical seizures can be provoked by DBS, for example in the “outlier” patient of the SANTE study. Onset of these seizures was simultaneous with the onset of DBS, and they differed from the patient’s habitual seizures in seizure duration and postictal period—both being significantly shorter (18). The seizure type, complex partial seizure [focal unaware, ILAE 2017 (17)] remained essentially the same. At the time of onset of DBS therapy and the occurrence of atypical seizures, the stimulation parameters were set to 5 V, 90 µs, and 145 Hz. During a time period of 48 h the outlier patient experienced 210 of these brief seizures, after which the stimulation amplitude was reduced to 4 V resulting in the cessation of these seizures. After seven months, the stimulation parameters were restored to their original values and after month 13, voltage was increased up to 9 V. However, no recurrence of the atypical seizure type could be witnessed.

The selection of stimulation parameters for each patient is a critical point in the commencement of DBS therapy. Generally, in DBS therapy, adjustments of the parameters produce a U-shaped response (19). This concept is explained by the observed change in symptom severity for better or worse as voltage, frequency or pulse width is increased from a subtherapeutic value. Gradual increase typically results in desired symptom alleviation. Increasing the parameters beyond this optimal point may, however, exacerbate the original symptoms or cause adverse effects. The location of electrodes in relation to the ANT is another important factor. Subtle variations in the location of the nucleus and differences in electrode locations even within the ANT can greatly affect treatment outcome (20). This also highlights the significance of proper selection of active contacts, even when the electrode placement is accurate. Clinical benefit is rarely achieved with contacts outside of or in the posterior region of ANT, likely due to the insulating effect of the white matter lamina surrounding the ANT and the more extensive connections of anterior region of the ANT (20). Findings in our patient are consistent with this knowledge, as can be seen in Figure 1. Only the topmost contacts—labeled 3 and 11—activate a sufficient number of neurons in the anterior ANT to achieve seizure suppression (Figure 1E).

This case highlights the potential for a different response to stimulation in the initial period, compared to subsequently. In our case, this shift in response appears to have occurred over a time frame of hours, in contrast to the SANTE outlier whose initial response remained the same for a couple of days. The SANTE study group restored the stimulation voltage to the original value only after seven months, whereas in the current case this was done the same day. The outlier patient received stimulation at 4 V long enough for plasticity of the brain to possibly play a major role in the altered treatment response. Our patient’s brain could not have undergone such anatomical changes in the short time window he received stimulation at lower voltage—meaning there has to be some other mechanism.

Suggested mechanisms of DBS include short, medium and long-term effects (21, 22). Due to the limited time frame during which our patient’s response to DBS changed, we assume that long term effects—such as synapse plasticity—have little to no significance in this case. Medium-term effects include alterations in the levels of neurotransmitter, but their role in the current case cannot be adequately assessed from the patient data available. Another proposed mechanism is the rapid disruption of pathologic oscillations with DBS pulses (19, 21, 22). The systems oscillator theory addresses physiologic and pathologic oscillations in brain networks (19). The basis of this theory is the existence of numerous groups of neurons, termed nodes, that act as oscillators across different networks. The nodes and oscillatory systems have their own frequencies, and their combined activity enables complex brain functions. Neurologic disorders are the result of flaws in this system and corruption of the information carried by neural networks. DBS at right parameters—frequency being the key one in this case—may achieve clinical benefits by resonating with these oscillations and correcting or amplifying the generated information or by simply overrunning the pathologic oscillations.

In this report, we have raised the question of how DBS can provoke seizures with parameters later leading to favorable treatment outcome. We have also reflected on the possible mechanisms responsible for these events, as well as on the possibility of considering findings similar to the ones described here and on the SANTE materials as clinical biomarkers in ANT-DBS therapy. At the very least, initial effects caused by stimulation should not be regarded outright negative, as they might be a part of the process in which DBS modifies brain networks.

## Concluding Remarks

Deep brain stimulation has successfully been used to treat multiple neurologic disorders, including epilepsy. Of the many challenges DBS faces as a treatment method for intractable epilepsy, the inability to reliably predict the treatment outcome is a major one. The discovery of an unambiguous biomarker for favorable clinical response to ANT-DBS would greatly aid patient selection for operation. This would also reduce the amount both surgery and stimulation-related adverse effects. Future research should emphasize this objective, whether by the means of electrophysiological methods or different imaging techniques. This case report offers an insight into a possible solution to this problem using electroencephalography findings as possible biomarkers. Our hypothesis is that favorable treatment outcome can be achieved despite initial stimulation induced side effects.

## Ethics Statement

This study was carried out in accordance with the recommendations of the Regional Ethics Committee of Tampere University Hospital with written informed consent from all subjects. All subjects gave written informed consent in accordance with the Declaration of Helsinki. The protocol was approved by the Regional Ethics Committee of Tampere University Hospital.

## Author Contributions

TN contributed significantly to the collection of patient information, conception and design of the study, and drafted the manuscript. HH and MT contributed to the collection of EEG data and assisted in interpreting it. KL and JP critically revised the earliest version of the manuscript and assisted in improving it with their expertise. KL provided us with the Suretune materials. All the authors revised the draft and gave their approval of the version to be published.

## Conflict of Interest Statement

KL and JP have received speaker and consultation fees from Medtronic. The reviewer LR and handling editor declared their shared affiliation.
